# Juvenile Polyposis of Infancy Presenting as Protein-Losing Enteropathy

**DOI:** 10.14309/crj.0000000000000542

**Published:** 2021-03-03

**Authors:** Natascha S. Sandy, Marcia Alessandra C. P. Cavalaro-Silva, Silvia R. Cardoso, Maria Angela Bellomo-Brandao

**Affiliations:** 1 Division of Gastroenterology, Hepatology and Nutrition, Department of Pediatrics, Hospital for Sick Children, University of Toronto, Toronto, Ontario, Canada; 2 Department of Surgery, Faculty of Medical Sciences, State University of Campinas, Campinas, São Paulo, Brazil; 3 Department of Pediatrics, Faculty of Medical Sciences, State University of Campinas, Campinas, São Paulo, Brazil

## CASE REPORT

**Figure 1.**
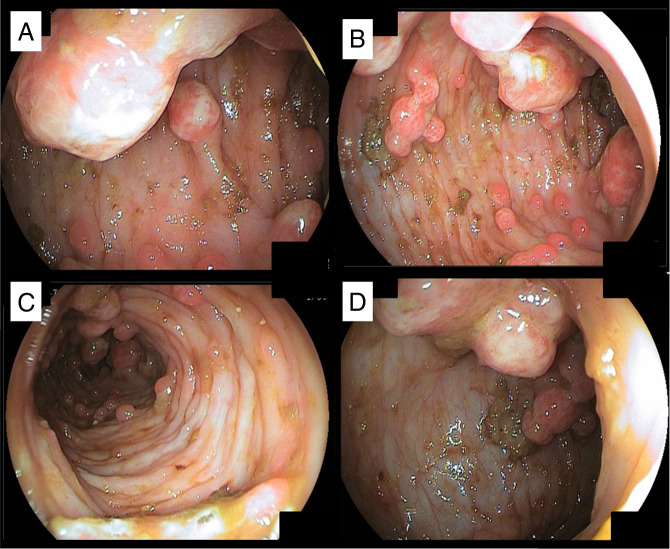
Severe diffuse colonic polyposis (A and B) ascending colon, (C) transverse colon, and (D) descending/sigmoid.

**Figure 2.**
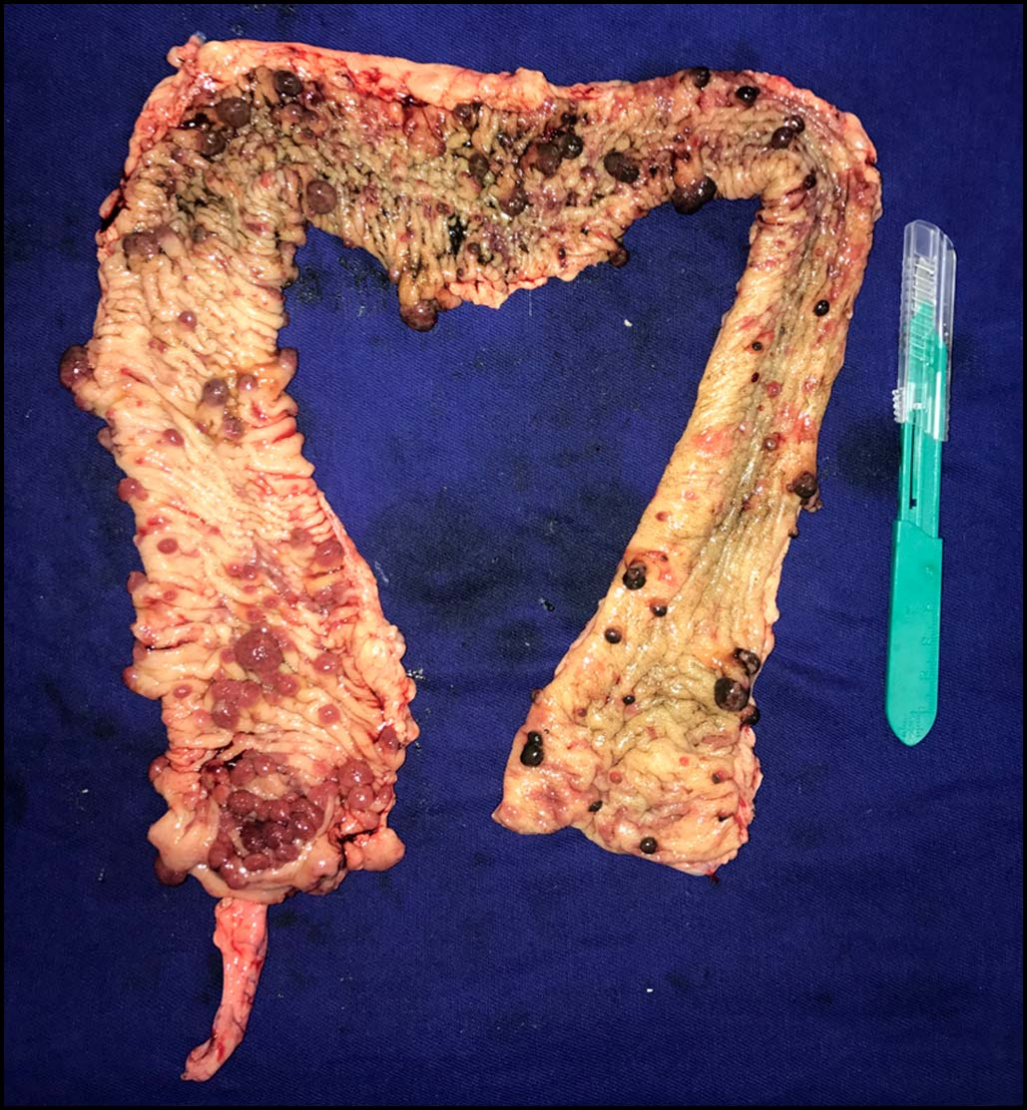
Macroscopic view of the colon after total colectomy.

A 20-month-old female was admitted with rectal bleeding and rectal prolapse with extrusion of a polyp. Her medical history was relevant for chronic diarrhea, abdominal distension since birth, delayed neuropsychomotor development, and refractory iron deficiency anemia. Physical examination revealed macrocephaly, skin pallor, severe malnutrition with significant muscle atrophy, and generalized edema. Laboratory workup demonstrated severe anemia and hypoalbuminemia. Subsequent abdominal scintigraphy with Tc99m marked albumin revealed intestinal protein loss to some degree in the small bowel and severe colonic loss. Endoscopic examination showed diffuse severe polyposis throughout the colon (Figure 1). The patient underwent total colectomy with the creation of an ileostomy which significantly improved the protein-losing enteropathy and allowed for nutritional rehabilitation (Figure 2).

Juvenile polyposis syndrome (JPS) is a rare autosomal dominant condition with an estimated prevalence of 1/100,000.^1^ Juvenile polyposis of infancy is considered the most severe form of JPS. This generalized, early-onset form of JPS manifests with diarrhea, anemia, gastrointestinal bleeding, rectal prolapse, intussusception, and protein-losing enteropathy—as illustrated in this case.^1,2^ Depending on the magnitude of protein loss, colectomy may be required to treat protein-losing enteropathy. The use of sirolimus has also been reported as a management strategy for juvenile polyposis of infancy.^3^

## DISCLOSURES

Author contributions: NS Sandy and MA Bellomo-Brandao wrote the manuscript. SR Cardoso and MACP Cavalaro-Silva provided images and revised the manuscript for intellectual content. MA Bellomo-Brandao is the article guarantor.

Previous presentation: This case was presented at the 17th Brazilian Congress of Pediatric Gastroenterology; September 29-October 1, 2018; Porto de Galinhas, Brazil.

Informed consent could not be obtained from the patient despite several attempts. All identifying information has been removed from this case report to protect patient privacy.
